# Major Incongruence and Occupational Engagement: A Moderated Mediation Model of Career Distress and Outcome Expectation

**DOI:** 10.3389/fpsyg.2019.02360

**Published:** 2019-10-18

**Authors:** Ji Geun Kim, Ki-Hak Lee

**Affiliations:** Department of Psychology, Yonsei University, Seoul, South Korea

**Keywords:** major incongruence, career distress, occupational engagement, career outcome expectation, moderated mediation model

## Abstract

This study investigated the possible mediation of career distress in the relationship between major incongruence and occupational engagement and whether this mediation depends on the degree of outcome expectation. Moderated mediation analysis was tested on a sample of 346 Korean undergraduate students. The results indicated that career distress mediated the relationship between major incongruence and occupational engagement. Moreover, the negative indirect effect of major incongruence on occupational engagement through career distress weakened as the level of outcome expectation increased. The significant mediation effect of career distress is meaningful given the evidence on the role of emotion in career adaptation. In addition, the significant moderation effect of cognitive evaluation and belief in the mediating relationship on career problem, career emotion, and career behavior is meaningful in that it provides insights in cognitive intervention that could be effective in career counseling.

## Introduction

One of the most important expectations that undergraduates have about the education they receive it that it meets the demands of their future career (Li et al., 2013). In many cases, undergraduates gain the knowledge and skills to enter the professional world through their course of study. Thus, the congruence between current academic major and desired job domain is bound to be closely related to undergraduates’ successful transition to their future career path (Wolniak and Pascarella, 2005). However, many undergraduates experience a mismatch between their current majors and their desired career paths for two main reasons: limited knowledge on which major is related to which career path upon university entrance, and choice of major without thorough consideration of how major choice may influence future career paths (Galotti, 1999; Shin et al., 2014). This study referred to the mismatch between academic major and desired career goal after graduation as “major incongruence” and explored its effects on the career adaptation of undergraduates.

Person-environment (P-E) fit theory (Muchinsky and Monahan, 1987) is the most widely used theory in studying undergraduates’ major incongruence. Among various P-E fit types, the agreement between one’s major and their desired career goal, or major congruence, can be explained by need-supplied (N-S) fit. N-S fit occurs when the environment satisfies an individual’s needs, desires, or preferences (Kristof, 1996). In other words, the environment is the major and the individual’s need is the desired career goal and the N-S fit indicate the agreement between the students’ desires and the rewards they receive in return for their study. In attempting to capture how one’s needs are fulfilled in one’s major, previous studies have taken a broader perspective in measuring major fit. Specifically, the needs for major include multidimensional concepts, such as academic goal and vocational career (Etzel and Nagy, 2016), which make it difficult to clarify which specific factor of major fit is related to outcome variables. Thus, in the present study, we focused on preparing for occupation, which is the number one goal that undergraduates have during college (Cuseo, 2005). We measured major fit with the congruence between the individuals’ current major and desired career path.

In studies on major fit, the measurement method is another factor that influences the relationship between fit and outcome (e.g., Wessel et al., 2008). These measurement methods include directly asking individuals their perceived fit and objectively comparing the traits of separately evaluated P and E to evaluate the fit (Kristof-Brown et al., 2005). Wessel et al. (2008) tested which of the objective fit measure and perceived fit measure better explain the relationship between major fit and outcome. As a result, perceived fit had a stronger relationship with affective major commitment and academic self-efficacy than the objective fit. Other studies have also supported a similar result in which perceived fit had stronger relationships with outcomes than objective fit (Kristof-Brown et al., 2005). Based on these findings, the concept of perceived fit has been actively used in the major fit literature; perceived major fit is associated with outcome variables such as adaptability, major change intention, academic performance, academic satisfaction, depression, class absenteeism, calling, and meaningful work (Schmitt et al., 2008; Li et al., 2013; Shin et al., 2014; Etzel and Nagy, 2016; Vahidi et al., 2016). These results have indicated that perceived major fit is an important factor affecting undergraduates’ attitude and behavior. Thus in the present study, we decided to measure the congruence between major and desired career goal via perceived fit.

Another point to note in the major fit literature is that most studies focus only on the relationship between major fit and outcomes. However, career adaptation is an ongoing process; thus, major fit can be formed through dynamic interaction between the individual and their environment (Culpepper, 2006; Vahidi et al., 2016). For undergraduates experiencing major incongruence, it is important to seek the help they need, gather information, and explore possibilities through challenges and experiences, to make the best choice in the *status quo* rather than suffer in frustration. Such career problem-solving experience can be a critical learning experience in their lives. Thus, studies on interventions that can lead the major incongruence experience into an adaptive one are necessary. In this study, we explored potential areas of intervention for major incongruence by identifying the mediator and the moderator in the relationship between major incongruence and adaptive behavior.

This study aimed to test a moderated mediation model in which the relationship between major incongruence and occupational engagement is mediated by career distress and moderated by outcome expectation. First, we posited that the negative effect of major incongruence on occupational engagement will be mediated by career distress, a career decision-related stress (*Hypothesis 1*). Adaptive career behaviors are required for individuals to overcome career challenges (Lent and Brown, 2013). For example, when individuals experience major incongruence, they need to engage in various career-related behaviors, such as re-explore their interests and aptitudes, seek information to realize their aspired for career, and attempt a double major program or changing majors. Adaptive occupational engagement occurs when the problem individuals perceive is at a minimal level (Lent et al., 2000; Lent, 2005). In other words, as individuals experience higher levels of major incongruence, they may engage in limited career behaviors. In addition, career issues entail strong affective factors (Krumboltz, 1993). Major incongruence has been associated with increased strain (Li et al., 2013). The negative emotion related to career issues also leads to a decrease in adaptive career behaviors (Emmerling and Cherniss, 2003). Thus, major incongruence may have a direct impact on the decrease in occupational engagement but also an indirect one on the decrease in occupational engagement via negative emotion that stems from career issues.

**FIGURE 1:**
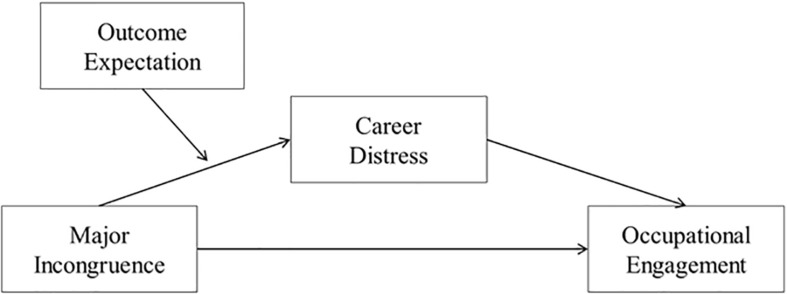
Theoretical model.

We also posited that the indirect effect of major incongruence on occupational engagement via negative emotion will be contingent on outcome expectation (*Hypothesis 2*). Outcome expectation is defined as the expectation on the outcome of a particular behavior (Bandura, 1986), which can be understood as the belief on receiving sufficient tangible or intangible compensation for one’s effort. High levels of outcome expectation have been shown to lower the perceived barriers related to career and increase the persistence of goal-oriented behavior (Lent and Brown, 2006). In other words, individuals who expect positive outcomes from their effort are more likely to perceive major incongruence as a solvable problem and thus continue their efforts in pursuing academic and career goals. Meanwhile, those who expect that their effort will not yield corresponding results will be stressed and feel listless, leading to poorer career behaviors. Thus, we aimed to investigate outcome expectation, or positive expectation for efforts, as a variable that buffers the indirect negative effect of major incongruence on adaptive behavior via negative emotion. We aimed to expand the current P-E fit model literature, which is limited to major fit and outcome relationship by examining a moderated mediation model, and then suggest implications for potential emotional and cognitive intervention areas in career counseling. The research model is shown in Figure 1.

### Major Incongruence, Career Distress, and Occupational Engagement

Choosing a major in college is one of the most important decisions students have to make (Porter and Umbach, 2006). However, the career counseling in Korea is mostly focused on helping students to earn high scores on college entrance exams to get into prestigious schools rather than helping them explore career options via various career activities (Lee, 2001; Hwang et al., 2006). Thus, Korean students tend to choose whichever major they can get into based on their college entrance exam scores (Lee, 2008) and only after they get into college, they actually start to explore career options and decide on their careers. This is problematic because changing major in Korea is particularly more difficult. Thus, misselected major is likely to lead to maladjustment in academic settings and overall college life and even to ineffective career preparatory behaviors which may result in difficulties in employment (Au, 2011). This is also reflected in the high percentage of those getting jobs that are unrelated to their field of study in Korea (50%) which is the highest among the 22 OECD countries (Montt, 2015).

Considering the mismatch between current major and desired career path as a factor that hinders undergraduates’ career adaptation and development, major incongruence is an important career barrier that is defined as events or conditions that make career progress difficult (Swanson and Woitke, 1997). The more career barriers are perceived negatively, career exploration behaviors directed at overcoming career barriers are limited (Luzzo and Jenkins-Smith, 1998). Moreover, those who perceive higher levels of career barriers are less likely to engage in career behaviors related to their career interest (Brown and Lent, 1996). Based on this rationale, we posited that undergraduates who experience major incongruence will be less involved in career-related activities. In a previous study on undergraduates, students experiencing difficulties because of major incongruence show passive career search attitudes (Kim, 2014).

Of the various career behavior constructs, occupational engagement has gained attention as an important concept in undergraduates’ adaptation in the rapidly changing work world (Khasawneh, 2011). In their trilateral model, Krieshok et al. (2009) have emphasized occupational engagement as an important behavioral component of the career decision-making process along with rationality and intuition. Specifically, occupational engagement is the foundation for adaptive career decision that balances rationality and intuition. Occupational engagement is defined as behavior that broadens career options and improves understanding of the world of work and oneself (Black, 2006). Specifically, in the trilateral model, occupational engagement is deemed as a continuing process of promoting career exploration and preparing for future career adaptation (Cox, 2008). In other words, occupational engagement can be seen as an open and opportunity-oriented behavioral variable that provides information and experience by getting deeply engaged in the career-related issue and overall life (Krieshok et al., 2009). Thus, occupational engagement is a concept that represents the continuous career behavior required in the process of finding career direction for undergraduates with major incongruence. Therefore, this study aimed to explore the areas of intervention that can alleviate the reduction of adaptive behavior caused by major incongruence by setting occupational engagement as an important outcome variable.

According to a qualitative study on major incongruence, the core experience reported are disappointment, depression, frustration, and stress, which are mostly emotional experiences (Ntemngweh, 2016). Despite such results, quantitative research on the experience of undergraduates’ major incongruence has focused on the cognitive and behavioral processes, without considering the emotional aspect. The role of emotions, which has often been disregarded in the career counseling field, has been gaining attention recently (Hartung, 2011; Puffer, 2011). Emotions influence the entire career decision-making process, including the number of career options considered, time and effort spent on career exploration, and manner of integrating information (Emmerling and Cherniss, 2003). Therefore, in the present study, the negative emotions related to major incongruence were considered as important variables that influence career adaptation and development. A subjective career distress scale that measures helplessness, depression, stress, and anxiety experienced in career decision making was used to measure the negative affect.

Hartung (2011) suggested that emotion could be a basis for explaining specific career behaviors. This rationale is based on the view that emotions resulting from responses to the environment are important motivators of future coping behaviors and that certain behaviors increase or decrease based on emotions (Lazarus, 1991). For example, the fear and anxiety associated with career decision making influence one’s intention to stay in an unsatisfactory current career (Emmerling and Cherniss, 2003). Thus, negative emotions caused by major incongruence might lead to a decrease in occupational engagement behaviors that facilitate career decision making. In the present study, based on the results of previous studies, we set up a mediating model that examined the effect of major incongruence on occupational engagement via negative emotions (*Hypothesis 1*).

### Outcome Expectation as a Moderator

Outcome expectation is the cognitive assessment of outcomes, such as hopes and beliefs following an action or effort (Bandura, 1997). Outcome expectation affects individuals’ perception of career barriers (Lent et al., 2000). Related studies have shown that the higher the expected career outcome, the less likely an individual is to perceive one’s difficulties as challenging (Betz and Voyten, 1997) and this is also supported by a Korean study (Shin and Kim, 2013). In considering major incongruence as a barrier that hinders the pursuit of a career goal, one’s positive outcome expectation of their effort will enable them to perceive the barrier as less threatening. Lent et al. (1994) asserted that outcome expectations are formed through various imitation experiences related to various educational and occupational activities. Thus, this study posited that interventions on outcome expectation will enable one’s cognitive reevaluation of major incongruence, which can buffer the negative effect of major incongruence on career development.

Outcome expectation also has been studied as a variable that functions to sustain and maintain career behavior (Betz and Voyten, 1997; Gore and Leuwerke, 2000). The motivation for human behavior stems from the interaction between desired outcomes and their feasibility (Rotter, 1966; Vroom, 1982). In other words, no matter how valuable a goal is, if the feasibility is close to zero, individuals will not be motivated, and vice versa (Nagengast et al., 2011). Applying to a case of major incongruence, if an individual believes that their desired career is difficult to achieve because of major mismatch, then they will not be motivated in their career. Meanwhile, if they can reframe the negative cognition, and think that they can achieve the desired career through effort, then they will be motivated to expand and integrate other competencies into their major.

With respect to outcome expectation and emotions, people with depression (Showers and Ruben, 1990) and high anxiety (Kelly and Shin, 2008) tend to have negative outcome expectations, including career outcomes. In other words, it may be difficult to have positive outcome expectations while being in negative emotional states. Thus, we posited a moderated mediation model in which the interaction between major incongruence and outcome expectation on the mediation path of emotional difficulties of major incongruence inhibits occupational engagement (*Hypothesis 2*).

## Materials and Methods

### Participants

The study’s sample consisted of 346 undergraduate students (females, 63%) enrolled in a large university in Seoul, Korea. All participants had the same ethnic background, and their age ranged from 18 to 29 years (M = 22.61 years, *SD* = 1.99 years). All students in general psychology classes were invited to participate in the study. They answered a survey via a university-based student research pool website introduced during the class and then received extra credit for participating. Participants accessing the online survey first read and agreed with informed consent procedures: explaining the purpose of the study, capability to discontinue the survey, and confidentiality. Completion of measures required approximately 15–20 min. We excluded incomplete responses and screened scores on all key measures for multivariate outliers with Mahalanobis Distance. The sample included students of all year levels and different majors: 39 (11.3%) freshmen, 129 (37.3%) sophomores, 67 (19.4%) juniors, and 111 (32.0%) seniors; 99 (28.6%) liberal arts, 62 (17.9%) business and economics, 57 (16.5%) engineering, 38 (11.0%) social sciences, 29 (8.4%) science, 25 (7.2%) music and sport, 11 (3.2%) medicine and dentistry, 11 (3.2%) international college, 8 (2.3) theology, and 6 (1.7) education.

### Measure

#### Major Incongruence

To measure the agreement between the major and desired career, participants were first asked to respond “Yes or No” to the following question, “Have you ever thought about which career path you would like to pursue?” 18 of those who responded “No” to this question were deleted. Those who responded “Yes” proceeded with the survey. Then, we used a single, face-valid item (“Is your academic major congruent with your desired future career?”) to measure major incongruence that was used in a previous study (Shin et al., 2014). Shin et al. (2014) have used “Yes or No” scale in their study, however, they pointed out that using a forced-choice method such as this, to dichotomize major incongruence would limit the scope of individual differences. Thus, they recommended that future studies change the response scale to enable measuring the degree of incongruence. Thus, we used a Likert-type scale. Participants indicated the extent to which they perceived major congruence on a nine-point Likert-type scale from 1 (not congruent at all) to 9 (strongly congruent). The item was reverse-scored for analysis; a higher score corresponded to higher incongruence. The usefulness and reliability of using a single item has been demonstrated (Wanous and Reichers, 1996; Robins et al., 2001). Several vocational variables have been assessed using single-item measures, such as major congruence, person-environment fit, and job satisfaction (Wanous et al., 1997; Lachterman and Meir, 2004; Shin et al., 2014).

#### Career Distress

To measure career distress, we used the 13-item Subjective Career Distress Subscale from Coping with Career Indecision scale (Larson et al., 1994), which assesses distress relating to career decision making and goal setting. We used the Korean version, which has been validated by Lee (2005). Sample items are “I often feel down or depressed about selecting a career” and “I feel stress or pressure in selecting a satisfying major and career.” Participants indicated their level of agreement with each item a five-point Likert response scale from 1 (strongly disagree) to 5 (strongly agree); higher scores represented more career distress. Larson et al. (1994) reported an internal consistency coefficient of 0.90. For the current sample, the internal consistency coefficient was 0.84.

#### Occupational Engagement

Occupational engagement was assessed using the Occupational Engagement Scale for Students (Cox, 2008), which represented behaviors college students engage in that help them gain knowledge on themselves, the world, and the relation between the self and the world. The Korean version of the Scale was translated and empirically reviewed by Jung (2011); the Korean version reflected the intentions of the original scale. The Scale consists of a 14-item single factor, and sample items are “I attend presentations or talks related to a career I might find interesting” and “I do lots of things that are interesting to me.” The statements are rated on a five-point Likert scale (1 = strongly disagree, 5 = strongly agree). Cox (2008) reported internal consistency of 0.85, and Jung (2011), 0.87. The alpha value for the current sample was 0.87.

#### Outcome Expectation

Outcome expectation was assessed using the Career Outcome Expectancies Scale (Betz and Voyten, 1997), which defines outcome expectation as the belief regarding the consequences of success in specific educational performance or career behaviors. We used the Korean version, validated by Yang (2006). This Scale is composed of the two factors of educational (five items) and career outcomes (four items). Sample items are “If I learn more about different careers, I will make a better career decision” and “If I get food grades, I will be able to have the career of my choice.” Responses were rated on a five-point Likert scale (1 = strongly disagree, 5 = strongly agree). Values of the internal consistency coefficient of the original scale were 0.77–0.79. Yang (2006) reported values of 0.77–0.79. The internal coefficient of the current sample was 0.81.

### Data Analyses

Data were analyzed using SPSS 23.0. First, the relationships between variables were examined by correlational analysis. Then, one-way ANOVA was conducted to test if there are significant differences among year in college and major. In estimating parameters of moderated mediation based on sample data, the normality of residuals should be assumed (Muller et al., 2005). Thus, next we tested the normality of residuals and as a result, the assumption was met (Kolmogorov-Smirnov test: *p* > 0.05). Fourth, the proposed theoretical model (Figure 1) was tested via simple and moderated mediation analyses using the SPSS mediation and moderated mediation macro developed by Hayes (2017). Using this analysis, we examined the direct and indirect effects of an independent variable (major incongruence) on a dependent variable (occupational engagement) via a mediator (career distress), and if the effect is conditional on the moderator (outcome expectation). As Aiken and West (1991) suggested, all continuous variables were mean-centered prior to the analyses to reduce the multicollinearity between main effects and interaction. Bias-corrected confidence interval (CI) using 5,000 bootstrapped resamples were generated for verifying conditional indirect effects. Point estimates were considered significant if the 95% CI did not contain zero. Missing data were imputed using the Expectation Maximization (EM) algorithm in SPSS (Enders, 2001).

## Results

### Descriptive Statistics, Correlation Analyses, and Analysis of Variance

Means, standard deviations, and inter-correlations among variables are presented in Table 1. An examination of the correlations showed that major incongruence was positively related to career distress (*r* = 0.29, *p* < 0.001) and negatively related to occupational engagement (*r* = −0.28, *p* < 0.001), whereas career distress was negatively correlated with occupational engagement (*r* = −0.28, *p* < 0.001). Thus, recognizing that one’s current major does not match one’s desired career can be associated with strong negative emotions. Moreover, perceiving major incongruence and experiencing negative emotions related to career decision making can be associated with a lack of career-related behaviors, such as gathering job-related information and gaining experience.

**TABLE 1:** Descriptive statistics and correlations of major incongruence, career distress, occupational engagement, and outcome expectation.

**Variable**	***M***	***SD***	**α**	**1**	**2**	**3**	**4**
1. Major incongruence	4.10	2.39		–			
2. Career distress	43.05	9.88	0.84	0.29^∗∗^	–		
3. Occupational engagement	45.25	9.35	0.87	–0.28^∗∗^	–0.28^∗∗^	–	
4. Outcome expectation	34.57	5.12	0.81	–0.14^∗∗^	0.01	0.17^∗∗^	–

**^∗∗^p* < *0.01.**

Outcome expectation was negatively correlated with major incongruence (*r* = −0.14, *p* < 0.001), positively correlated with occupational engagement (*r* = 0.17, *p* < 0.001), and showed no statistically significant correlation with career distress (*r* = 0.01, ns). In other words, high levels of positive belief in one’s career decision and educational performance are associated with low levels of major incongruence, high levels of career behaviors, and no significant relationship with negative affect.

Age/year in college and major have been studied as important variables in studying college students’ career development (Patton et al., 2003). However, the findings on the relationship among age/year in college, major and career barrier, career decision, career behavior, and career satisfaction are mixed (Earl and Bright, 2003; Uthayakumar et al., 2010; McIlveen et al., 2013; Park and Ahn, 2016). Therefore, one-way ANOVA analysis was carried out to determine whether there was a difference in measures according to grade and academic majors to more accurately test the relationships among the variables. Results of one-way ANOVA indicated no significant main effects of grade on major incongruence [*F*(4, 341) = 0.56, *p* = 0.690], career concern [*F*(4, 341) = 0.41, *p* = 0.804], occupational engagement [*F*(4, 341) = 1.74, *p* = 0.141], and outcome expectation [*F*(4, 341) = 0.97, *p* = 0.426], and also no significant main effects of academic major on major incongruence [*F*(9, 336) = 0.49, *p* = 0.879], career concern [*F*(9, 336) = 0.51, *p* = 0.864], occupational engagement [*F*(9, 336) = 1.053, *p* = 0.397], and outcome expectation [*F*(9, 336) = 1.283, *p* = 0.245]. There were no differences among study variables based on participant characteristics thus, year in college and major were not included as controls.

### Simple Mediation Analyses

As shown in Table 2, results of the simple mediation model indicated that major incongruence was significantly related to career distress (*B* = 1.21, *t* = 5.66, *p* < 0.001). The relationship between career distress and occupational engagement was also significant (*B* = −0.20, *t* = −4.05, *p* < 0.001), controlling for major incongruence. Major incongruence was negatively associated with occupational engagement when controlling for career distress (*B* = −0.84, *t* = −4.04, *p* < 001). An indirect effect of major incongruence on occupational engagement via career distress was significant (*B* = −0.25, *p* < 0.001). Results of the bootstrapping also confirmed the indirect effect (95%CI: [−0.43, −0.11], not containing zero).

**TABLE 2:** Regression results for simple mediation.

	***B***	***SE***	***t***	***p***
Occupational engagement regressed on major incongruence	–1.084	0.203	–5.336	0.000
Career distress regressed on major incongruence	1.208	0.214	5.656	0.000
Occupational engagement regressed on career distress, controlling for major incongruence	–0.203	0.050	–4.051	0.000
Occupational engagement regressed on major incongruence, controlling for career distress	–0.838	0.208	–4.035	0.000

**Bootstrapped indirect effects Of Major Incongruence on Occupational Engagement via Career Distress**				

	***B***	**Boot SE**	**Boot LLCI**	**Boot ULCI**
Career distress	–0.245	0.086	–0.455	–0.111

**B, unstandardized coefficients; SE, standard error; LLCI and ULCI, lower level and upper level of the bias-corrected 95% bootstrap confidence interval. N* = *5,000 bootstrapping resamples.**

### Moderated Mediation Analyses

**FIGURE 2:**
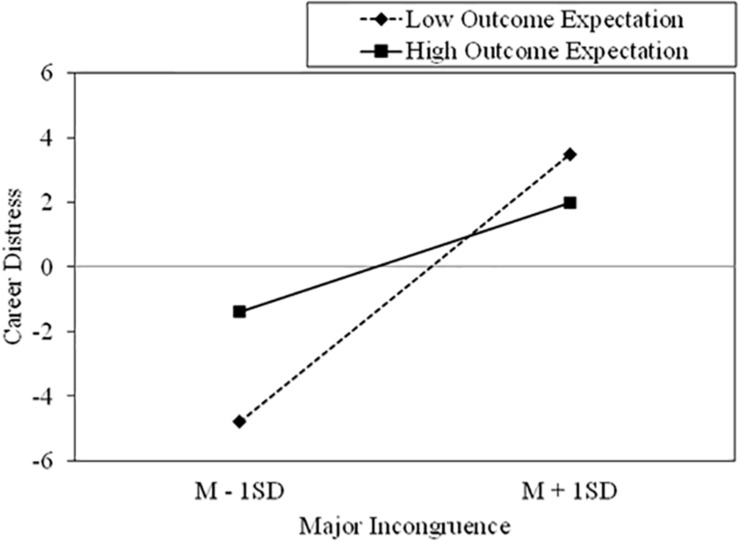
Interaction effect of outcome expectation between major incongruence and career distress. Unstandardized regression coefficients are used.

Moderated mediation analysis established whether an indirect effect occurred from major incongruence to occupational engagement via mediation of career distress, and if the effect was conditional on the moderation of outcome expectation. We hypothesized that participants with higher scores on outcome expectation would show a weaker relationship between major incongruence and career distress compared with those with lower scores. Table 3 shows the results of the moderated mediation model. The effect of the interaction between major incongruence and outcome expectation on career distress was statistically significant (*B* = −0.10, *t* = −2.53, *p* < 0.05). Figure 2 illustrates the interaction at high (+ 1 *SD*) and low (−1 *SD*) levels of major incongruence and outcome expectation. The plots indicated the interaction effect between major incongruence and outcome expectation on career distress; for students having lower levels of outcome expectation, there was a stronger positive relationship between major incongruence and career distress compared with students having higher levels of outcome expectation.

**TABLE 3:** Regression results for conditional indirect effect.

	**Career distress**					
	***B***	***SE***	***t***	***p***	**LLCI**	**ULCI**

Major incongruence (MI)	1.223	0.214	5.711	0.000	0.802	1.644
Outcome expectation (OE)	0.093	0.100	0.928	0.354	–0.104	0.289
MI × OE	–0.100	0.040	–2.533	0.012	–0.178	–0.023

	**Occupational engagement**					
	***B***	***SE***	***t***	***p***	**LLCI**	**ULCI**

Major incongruence	–0.838	0.208	–4.035	0.000	–1.245	–0.430
Career distress	–0.203	0.050	–4.052	0.000	–0.302	–0.105

	**Conditional indirect effect at specified values of outcome expectation**					
	***B***	**Boot SE**		**Boot LLCI**	**Boot ULCI**	

-1 SD (-5.118)	–0.353	0.114		–0.611	−0.154	
M (0.000)	–0.249	0.083		–0.436	−0.105	
+1 SD (+5.118)	–0.144	0.087		–0.351	−0.014	

	**Index of moderated mediation**					
	**Index**	**Boot SE**		**Boot LLCI**	**Boot ULCI**	

Career distress	0.020	0.011		0.003	0.049	

	**Conditional indirect effect at 10th, 25th, 50th, 75th, 90th percentiles values of outcome expectation**					
	***B***	**Boot SE**		**Boot LLCI**	**Boot ULCI**	

10th	–0.382	0.126		–0.684	−0.175	
25th	–0.321	0.101		–0.556	−0.150	
50th	–0.240	0.081		–0.427	−0.107	
75th	–0.199	0.079		–0.397	−0.076	
90th	–0.097	0.102		–0.341	0.065	

**B, unstandardized coefficients; SE, standard error; LLCI and ULCI, lower level and upper level of the bias-corrected 95% bootstrap confidence interval. N* = *5,000 bootstrapping resamples.**

The conditional indirect effect of major incongruence on occupational engagement through career distress at the values of outcome expectation was analyzed when the scores of outcome expectation were the sample mean and ± 1 *SD*. The results revealed that all of the three conditional indirect effects were significantly negative, and bootstrap CIs supported these results (see Table 3). Although the results indicated that the interaction effect between major incongruence and outcome expectation influenced occupational engagement through career distress, a more detailed examination of the conditional indirect effects model was needed. As shown in the bottom section of Table 3, when the range of outcome expectation was expanded, the negative effect turned out to be not significant (i.e., when the mean-centered scores of career-related outcome expectation was 5.91, the indirect effect was not significant). Thus, high major incongruence had no effects on decreasing occupational engagement through career distress when the score on outcome expectation was very high. This significant indirect effect means that outcome expectation attenuates the mediation effect of career distress between major incongruence and career engagement, consistent with the hypothesis.

## Discussion

According to studies on P-E fit conducted in the education field, major fit is an important factor that influences academic, career, psychological difficulties that college students face (Culpepper, 2006; Schmitt et al., 2008; Wessel et al., 2008; Li et al., 2013; Etzel and Nagy, 2016; Vahidi et al., 2016). Undergraduates perceive major fit in various areas, such as their vocational interest, value, and goals. Particularly, more attention needs to be given to the congruence between major and desired career as it affects both individuals’ career development and higher educations’ productivity. However, very few studies have focused on the discrepancy between major and desired career as major fit in career development studies. Previous studies have only focused on the relationship between major fit and outcomes, and are limited in providing implications for potential interventions. Thus, we aimed to investigate a moderated mediation model in which major incongruence indirectly affects occupational engagement through career distress, in a mediation moderated by outcome expectation. We hoped that the results of this study expand the currently limited P-E fit model and enable exploration of potential intervention areas for major incongruence.

The results of the study are as follows. First, career distress partly mediated the relationship between major incongruence and occupational engagement (*Hypothesis 1*). In other words, the major incongruence level was related to career decision-related stress and listlessness, and this negative emotion experience was related to low adaptive career behavior. This result is meaningful in that it supports the importance of emotion in career adaptation, and is in line with previous studies that identified a relationship between emotion and career-related behaviors. For example, Kelly and Shin (2008) found that negative emotions and anxiety restricted career search and resulted in career undecidedness. In addition, those with high negative emotions use avoidant coping strategies to cope with their environment (Brown et al., 2012). The results of this study suggest that career counselors need to have the ability to understand the relationship between negative emotions and career behaviors when dealing with major incongruence problems.

We also observed a significant moderating effect of outcome expectation in the relationship between major incongruence and career distress. Further, in verifying the statistical significance of the moderated mediation model, which integrated the mediation and moderation models, we noted that the indirect effect of career distress on the relationship between major incongruence and occupational engagement was moderated by outcome expectation. When the significance of the mediating effect was extended beyond ±1 SD, the mediating effect was no longer significant at the very high level of outcome expectation. In other words, if students firmly believed that their academic and career endeavors will produce positive results, then the negative indirect effect of major incongruence reducing occupational engagement via career distress would no longer be significant. These findings support previous researches that individuals with a positive outlook on outcomes are less likely to perceive career issues as barriers and are more motivated to engage in beneficial behaviors (Betz and Hackett, 1997; Nagengast et al., 2011). This result suggests the importance of confirming in career counseling cognitive appraisal and belief in individuals who experience major incongruence.

### Theoretical and Practical Implications

The theoretical and practical implications of this study are summarized as follows. First, the present study expanded the existing P-E fit model that focused on the relationship between major fit and outcomes, and identified areas of intervention for major incongruence by testing mediators and moderators. With the rapid change in the work environment and uncertainty in the economy, continuing adaptive career behavior to cope with career crisis is important (Lent and Brown, 2013). In this study, we set occupational engagement as the adaptive career behavior variable that is required for students who experience major incongruence in deciding their career paths. Particularly, Korean students mostly focus on college entrance exams rather than exploring various careers until high school due to the Korean education system. They only start to explore career options once they are in college. Thus, career behaviors that involve deeply learning about the work world and themselves are important behavioral traits for Korean undergraduates. Previous studies have also supported this notion; occupational engagement has helped Korean undergraduates to pursue clear career goals (Kim et al., 2014) and improve on career adaptability (Kim and Lee, 2018). However, previous studies on major incongruence have focused only on the negative effects of major incongruences, neglecting the variables related to adaptive career behavior that are required for resolving this career issue. To address this limitation, the present study explored the role of affective and cognitive variables in the relationship between major incongruence and adaptive career behavior. As such, we have provided an initial ground on which the previously studied major fit and outcome relations may be expanded in future studies.

Second, the role of emotions in the career adaptation process found in the mediation model should be considered more importantly in future career research and counseling. According to Emmerling and Cherniss (2003), career counseling should be a process in which clients gain insight on how emotions influence career decision-related behavior, including risk avoidance, impulsive decision making, and way of processing information. Clients should be guided to make career decisions using this knowledge. For example, if a client experiencing a major incongruence fails to adapt to the major or seek alternatives, the counselor can help the client express their negative emotions from the mismatch and understand how these negative emotions affect their current behavior. In other words, if negative emotions are hindering or limiting career behavior, counselors can help clients cope with current situations in more adaptive ways by helping them to deal with negative emotions.

Third, through the moderated mediation model, we found the important role of individuals’ cognitive appraisal and beliefs during a career crisis. In a qualitative study, students who overcame major incongruence reported considering the failure in a major selection process as a new opportunity to learn and explore new fields (Kim, 2014). In other words, they discarded the cognitive framework of considering the past as negative and used the experience as a driving force to move forward. In addition, previous research has reported that majors and job disagreements do not always negatively influence wage or job satisfaction (Allen and Van der Velden, 2001; Montt, 2015). Thus, mismatch between major and desired career does not always have to be viewed in a negative way. It could be turned into an opportunity for learning and growth, if one overcomes this process well. Therefore, to help students with major incongruence in career counseling, counselors should try to lower students’ negative perception of major incongruence and improve their expectation of outcomes. Particularly, if students have inaccurate or negative outcome expectations regarding their efforts toward academics or their major, counselors could intervene by helping them reappraise the misperception.

### Limitations and Future Research Directions

The limitations of this study and suggestions for future research are as follows. First, the level of discrepancy between major and desired career path perceived by the individual was measured with one item. Although useful for measuring various career variables, including major incongruence, a single question may be limited in that other aspects of the incongruence are not considered. Thus, it would be necessary to test if the supported relations between major incongruence, career behavior, negative emotion, and outcome expectation can be applied to other areas, such as interests and aptitude. As individual perceptions of major incongruence can be diverse, specific studies will help counselors in developing effective intervention strategies in varying circumstances.

Second, this study assumed causality between major incongruence, career distress, and occupational engagement based on related theory and previous studies. However, careful interpretation of causal relationship is needed because it was tested using only cross-sectional data. All variables were measured using self-reported tools; thus, it was not possible to dismiss completely the possibility that the participants gave biased responses or defended themselves. Therefore, in future research, it will be necessary to consider alternative methods of measurement, such as the use of observation, interview, or behavioral indices, and to design a longitudinal study that tests the linear causal relationship model of career adaptation and development process for major incongruence.

Third, in future studies, it is necessary to investigate the actual experiences of students with major incongruence in their transition to the career world. Currently, universities and groups offer a variety of education and services to help university students choose their major. However, afterward, students experiencing mismatch face a lack of support. To promote social interest, researchers should study the effect of major incongruence on career adaptation and development from the viewpoint of the entire life-span perspective. Further, these results should be used to provide practical and effective help by developing and applying programs that can help in the career adaptation and development of undergraduates with major incongruence.

## Conclusion

This study explored the negative effects of the mismatch between one’s major and desired career path on career-related emotion and career behavior and furthermore tested whether the positive expectation of the result of individual effort plays a role in buffering the negative influence of the major incongruence. Our findings provide important theoretical and practical implications for career counseling. Future studies on major incongruence should expand the existing P-E fit model, which focuses only on the relationship between major fit and outcome. Counselors and professionals should focus more on major incongruence for the undergraduates’ successful transition to the workforce, as well as the adaptation and development of their career from the perspective of life-long career path.

## Data Availability Statement

The datasets generated for this study are available on request to the corresponding author.

## Ethics Statement

The studies involving human participants were reviewed and approved by the Review Committee (DRC) at the Department of Psychology, Yonsei University. The patients/participants provided their written informed consent to participate in this study.

## Author Contributions

All authors listed have made a substantial, direct and intellectual contribution to the work, and approved it for publication.

## Conflict of Interest

The authors declare that the research was conducted in the absence of any commercial or financial relationships that could be construed as a potential conflict of interest.
